# Ponatinib Induces Vascular Toxicity through the Notch-1 Signaling Pathway

**DOI:** 10.3390/jcm9030820

**Published:** 2020-03-18

**Authors:** Rosalinda Madonna, Damiana Pieragostino, Maria Concetta Cufaro, Vanessa Doria, Piero Del Boccio, Martino Deidda, Sante Donato Pierdomenico, Christian Cadeddu Dessalvi, Raffaele De Caterina, Giuseppe Mercuro

**Affiliations:** 1 Institute of Cardiology, University of Pisa, 56124 Pisa, Italy; rosalinda.madonna@unipi.it; 2 Department of Internal Medicine, Cardiology Division, McGovern School of Medicine, University of Texas Health Science Center at Houston, Houston, TX 77030, USA; 3 Department of Medical, Oral and Biotechnological Sciences, University ‘‘G. D’Annunzio’’ of Chieti-Pescara, 66100 Chieti, Italy; dpieragostino@unich.it; 4 Analytical Biochemistry and Proteomics Laboratory, Center for Advanced Studies and Technology (CAST), University “G. D’Annunzio” of Chieti-Pescara, 66100 Chieti, Italy; maria.cufaro@unich.it (M.C.C.); piero.delboccio@unich.it (P.D.B.); 5 Department of Pharmacy, University ‘‘G. d’Annunzio’’ of Chieti-Pescara, 66100 Chieti, Italy; 6 Institute of Cardiology, University “G. d’Annunzio” Chieti-Pescara, 66100 Chieti, Italy; dvanessa@libero.it (V.D.); pierdom@unich.it (S.D.P.); 7 Department of Medical Sciences and Public Health, University of Cagliari, 09042 Cagliari, Italy; martino.deidda@tiscali.it (M.D.); cadedduc@unica.it (C.C.D.)

**Keywords:** tyrosine kinase inhibitors, ponatinib, vascular toxicity, Notch-1

## Abstract

Ponatinib, a third-generation tyrosine kinase inhibitor (TKI), is the only approved TKI that is effective against T315I mutations in patients with chronic myeloid leukemia (CML). Specific activation of Notch signaling in CML cells by ponatinib can be considered as the “on-target effect” on the tumor and represents a therapeutic approach for CML. Nevertheless, ponatinib-induced vascular toxicity remains a serious concern, with underlying mechanisms being poorly understood. We aimed to determine the mechanisms of ponatinib-induced vascular toxicity, defining associated signaling pathways and identifying potential rescue strategies. We exposed human umbilical endothelial cells (HUVECs) to ponatinib or vehicle in the presence or absence of the neutralizing factor anti-Notch-1 antibody for exposure times of 0–72 h. Label-free proteomics and network analysis showed that protein cargo of HUVECs treated with ponatinib triggered apoptosis and inhibited vasculature development. We validated the proteomic data showing the inhibition of matrigel tube formation, an up-regulation of cleaved caspase-3 and a downregulation of phosphorylated AKT and phosphorylated eNOS. We delineated the signaling of ponatinib-induced vascular toxicity, demonstrating that ponatinib inhibits endothelial survival, reduces angiogenesis and induces endothelial senescence and apoptosis via the Notch-1 pathway. Ponatinib induced endothelial toxicity in vitro. Hyperactivation of Notch-1 in the vessels can lead to abnormal vascular development and vascular dysfunction. By hyperactivating Notch-1 in the vessels, ponatinib exerts an “on-target off tumor effect”, which leads to deleterious effects and may explain the drug’s vasculotoxicity. Selective blockade of Notch-1 prevented ponatinib-induced vascular toxicity.

## 1. Introduction

Chronic myeloid leukemia (CML) is a myelo-proliferative disease affecting primitive hematopoietic progenitor cells [1,2,3]. The best drugs for the treatment of CML are the Abelson-Breakpoint Cluster Region (BCR-ABL) tyrosine kinase (TKIs) inhibitors. Ponatinib (trade name: Iclusig, from Incyte Biosciences International Srl, Epalinges, Switzerland), a third generation TKI, allows inhibition of theT315I mutation of BCR/ABL [4]. The treatment goal for CML is no longer palliation or prolongation of survival, but instead is discontinuation of therapy and cure [2,5,6]. In fact, the overall survival of CML patients who respond to TKIs inhibitors is close to that of the healthy population, and the response in many patients is very profound, making it possible to consider stopping their treatment [6]. However, recent studies have shown a significant increase in the incidence of cardiotoxicity and vascular adverse events (VAEs) in patients treated with ponatinib and nilotinib, especially with regard to increased arterial blood pressure, venous thrombosis, progressive atherosclerosis with coronary artery disease (CAD) and peripheral arterial obstructive disease (PAOD) [7,8,9,10]. Ponatinib-induced VAEs are not rare, and may occur in up to 20%–42% of patients receiving the 45 mg/daily dose [11,12]. The exact etiology of VAEs is not clear, especially with regards to the responsibility of the drug in the onset of them. Vascular toxicity associated with ponatinib treatment might be a result of the direct effects of this drug on vascular endothelial cells and their progenitor cells. CAD and PAOD are closely associated with endothelial damage, which is the result of an imbalance between vascular damage and vascular repair. Endothelial dysfunction (one of the earliest steps of atherogenesis) and dysregulation of endothelial progenitor cells (EPCs) are key steps for such an imbalance. Among TKIs, nilotinib [13], but not imatinib, has been shown to promote the expression of proatherogenic adhesion molecules (CAM) on human umbilical endothelial cells (HUVECs), including ICAM-1 (CD54), VCAM-1 (CD106) and E-Selectin (CD62E). Thus, different TKIs have different clinical vascular safety profiles that could reflect different pathogenetic mechanisms. Whether ponatinib induces endothelial dysfunction (i.e., the expression of CAM in endothelial cells) is not known.

The Notch-1 signaling involves a bi-molecular interaction between receptor and ligand and influences many aspects of cell specification in the developing and in the adult. Multiple cell fate decisions are influenced by Notch-1, including differentiation, proliferation, apoptosis, migration and angiogenesis. With such a central role in cell fate decisions, the abnormal function/expression of Notch-1 is related to several common diseases, including cancers. Notch-1 has been shown to have both tumor suppressive and tumorigenic function in different contexts [14] and the level of expression has also been suggested to influence the degree of malignancy in a dose-dependent manner [15]. Thus, specific activation of Notch signaling in CML cells by ponatinib can be considered as the “on-target effect” on the tumor and represents a therapeutic approach for CML.

We hypothesized that ponatinib-induced vascular toxicity may result from direct effects of this drug on vascular endothelial cells and on its progenitor cells. Mechanistically, we hypothesized that ponatinib inhibits prosurvival and increases senescence in endothelial cells via the Notch-1signaling pathway, leading to endothelial apoptosis and dysfunction. We examined the specific expressional signatures of endothelial cells exposed to ponatinib by performing proteomic analysis. The endothelial effects of ponatinib could be blunted by the administration of neutralizing factor anti-Notch-1 antibody, which increased prosurvival signaling and inhibited apoptosis in endothelial cells.

## 2. Materials and Methods

### 2.1. Cell Cultures

Human umbilical vein endothelial cells (HUVECs) were harvested enzymatically from umbilical cords of healthy volunteer donors with Type II collagenase (Worthington Biochemical Corporation, Lakewood, NJ, USA) 0.1 mg/mL and propagated as described [16]. Purity of cultures (>90%) was evaluated by using von Willebrand factor immunostaining. At confluence, cells were re-plated on 1.5% gelatin-coated flasks and used at 20,000 cells/cm^2^. Cells were used within passage 4 after primary cultures. The study was approved by our institutional review board and carried out in accordance with the Declaration of Helsinki as revised in 2000. Informed consent was given by the persons donating umbilical cords. The endothelial cell constitutive antigen detected by the E1/1 antibody was assessed in selected experiments.

### 2.2. EPC Isolation from Peripheral Blood Mononuclear Cells

Peripheral blood (PB) mononuclear cells (PBMNCs) were isolated from 12 mL of PB harvested from healthy volunteer donors. Blood underwent gradient centrifugation using Ficoll-Paque PLUS (Amersham, Buckinghamshire, United Kingdom). 12 mL of PB (contained in 4 EDTA-vacutainers) were mixed with one part of Phosphate Buffered Saline (PBS). An equal volume of Ficoll was placed in a 50 mL falcon tube and blood-PBS mixture was carefully stratified onto Ficoll. The tube was centrifuged at 400 *g* or 1600 rpm at 20° for 35 min. Three layers were obtained at the end of centrifugation: a. an upper layer containing Plasma + PBS; b. a middle layer containing monocytes and lymphocytes; c. a lower layer containing Ficoll, neutrophils and erythrocytes. The middle layer was withdrawn and placed in a 50 mL falcon tube. A total of 25 mL of cold PBS was added and centrifuged at 1500 rpm for 5 min. The pellet was resuspended in 30 mL PBS/5% FCS, centrifuged at 1500 rpm for 5 min, washed again, then used for CFU-EC (colony forming units-endothelial cells or CFU-Hill) isolations.

### 2.3. CFU-EC Isolation and Quantification

CFU-EC were cultured using the EndoCultTM Liquid Medium kit (Stem Cells Inc., Vancouver, Canada), according to the manufacturer’s instructions. Briefly, 5 × 10^6^ PBMNC were plated onto fibronectin-coated six-well plate in duplicate and incubated in EndoCult TM medium for two days at 37 °C, 5% CO_2_ with 95% humidity. After 48 h, non-adherent cells were collected and transferred into individual 5 mL tubes. Afterwards, 1 × 10^6^ cells of non-adherent cells were re-plated in each well of fibronectin-coated 24-well plates and cultured in EndoCult TM medium for additional 5 days. These cells organize in small clusters of central rounded cells with radiating spindle-shaped cells that disappear from 10–14 days onwards. At day 5 after plating in fibronectin-coated 24-well plates, clusters were counted in 8 randomly selected high-power fields.

### 2.4. Cell Culture Treatments

CFU-ECs and HUVECs were treated with decreasing concentrations of ponatinib dissolved in Dimethyl sulfoxide (DMSO) up to the concentration compatible with cell maintenance in the cell cycle tested by cell proliferation assay (1.7 nM corresponding to clinically used oral doses of 45 mg), accordingly with a time course from 0 to 72 h. The controls were treated with the vehicle (DMSO). In parallel experiments, CFU-ECs and HUVECs were treated with 1.7 nM ponatinib + 1 μg/mL neutralizing factor anti-Notch-1 antibody (R&D system, Minneapolis, MI, USA) [17]. Primary endpoints were subjected to clonogenesis from CFU-ECs by evaluating the number of early colonies, senescence, apoptosis, cell survival and proliferation and tubulization of HUVECs, as detailed below.

### 2.5. Cell Proliferation Assay

The effect of ponatinib on HUVECs proliferation was measured with the CyQUANT NF Cell Proliferation Assay Kit (Life Technologies, Grand Island, NY, USA), measuring cellular DNA content accordingly with the vendor’s protocol. Briefly, 5 × 10^3^ HUVECs were seeded in a 96-well plate for 24 h followed by treatment with 1.7 nM ponatinib or DMSO or 1.7 nM p+ anti-Notch-1 antibody for 17 h. Then HUVECs were incubated with 1× dye binding solution at 37 °C for 30 min in the dark. Fluorescence was detected with a microplate reader (Perkin Elmer, Milano, Italy) with excitation at 485 nm and emissions at 530 nm.

### 2.6. Label Free Proteomics

To analyze the effects of ponatinib on the specific expressional signatures in endothelial cells, shotgun proteomics analyses were performed, accordingly with methods already in place in our laboratory [18,19]. HUVECs were treated with 1.7 nM of ponatinib or DMSO or 1.7 nM of ponatinib + anti-Notch-1 antibody for 17 h. At the end of treatments, samples were prepared according to the Filter Aided Sample Preparation (FASP) method. Briefly, cellular pellets were lysed by sonication in a lysis buffer (urea 6 M in 100 mM Tris/HCl, pH 7.5) and after centrifugation of cell debris, the supernatants were assayed for protein concentration through Bradford assay (Bio-Rad, Hercules, CA, USA) using Bovine Serum Albumin (BSA, Sigma-Aldrich, St. Louis, MI, USA) standard for the calibration curve. Next, 50 µg of proteins was digested for each treatment by using trypsin (Promega, Madison, WI, USA). For protein label free identification and quantification, tryptic peptides from each sample were analyzed in triplicate with LC-MS/MS using a Proxeon EASY-nLCII (Thermo Fisher Scientific, Milan, Italy) chromatographic system coupled to a Maxis HD UHR-TOF (Bruker Daltonics GmbH, Bremen, Germany) mass spectrometer as already described [20].

### 2.7. Proteomics Data Processing

Proteomics raw data were processed using a free computational platform, MaxQuant version 1.6.6.0 (Max-Planck Institute for Biochemistry, Martinsried, Germany). Peak lists, generated in MaxQuant, were searched using Andromeda peptide search engine against the UniProt database (released 2018_04, taxonomy *Homo Sapiens*, 20,874 entries) supplemented with frequently observed contaminants and containing forward and reverse sequences. Carbamidomethylation of cysteines (C) was defined as fixed modification, while oxidation of methionines (M), deamidation of asparagines (N) and glutamines (Q) were set as variable modifications. Mass tolerances were set by default to 0.07 Da in the first search and 0.006 Da in the main search, while TOF MS/MS match tolerance was set to 0.05 Da. A retention time tolerance of 0.7 min was used to align any time shift in acquisition between samples. False discovery rate (FDR) at the protein level was set at 2%, on the contrary at peptide level was set at 1%. Intensity-based absolute quantification (iBAQ) was used to quantify protein abundance in each sample. Bioinformatics analysis was performed with Perseus version 1.6.2.3. Variability between the two different cellular treatments was reported as density plot with Pearson correlation of mean iBAQ transformed to log_2_ scale. In order to define the proteins differentially expressed between the HUVECs exposed to 1.7 nM ponatinib, or 1.7 nM ponatinib + anti-Notch-1, a univariate statistical analysis was performed with a *p*-value threshold of 0.05. Results were visualized as Volcano Plot. Differentially expressed proteins were analyzed using Ingenuity Pathway Analysis (IPA), (Ingenuity Systems, Mountain View, CA, USA). Through the Pathways analysis and the Gene Ontology, it is possible to identify the metabolic pathways and the secondary genes/proteins inhibited and/or stimulated for a specific phenotype and consequently classify potential effectors molecules and/or a pharmacological target.

### 2.8. Tube Formation Assay

In vitro HUVEC functionality in response to ponatinib was evaluated by using the tubulization assay. Specifically, tubulization (in terms of network area and number) was taken to represent a surrogate indicator of the HUVEC-mediated angiogenic capacity. Matrigel (BD Biosciences, San Jose, CA, USA) was thawed overnight at 4 C. Each well of a pre-chilled 24-well plate was coated with 300 μL matrigel and incubated at 37 C for 1 h. HUVECs (1.3 × 10^5^ cells) were added in 300 μL medium with 1.7 nM ponatinib or DMSO or 1.7 nM ponatinib + anti-Notch-1 antibody for 17 h. After incubation, the endothelial cell tube formation was assessed with Leica inverted microscope (Leica, Wetzlar, Germany). Images were taken from 8 regions in each well. Tubular structures were quantified by using the Angiogenesis Analyzer with Image J (version 1.49, National Institutes of Health, Bethesda, MDUS).

### 2.9. Senescence-Associated β-galactosidase Assay

The effect of ponatinib on HUVEC senescence was evaluated by SA-b-gal Activity Senescence-Associated-β-Galactosidase Staining (Cell Biolabs, Inc, San Diego, CA, USA) accordingly with the vendor’s protocol. Briefly, HUVECs were plated at 2 × 10^5^ cells per well in a 6-well dish, grown overnight and then treated with 1.7 nM ponatinib or DMSO or 1.7 nM ponatinib + anti-Notch-1 antibody for 17 h. The next day, cells were rinsed with sterile PBS, fixed with 0.5% glutaraldehyde at room temperature for 15 min and then rinsed with sterile PBS. Cells were stained overnight with fresh senescence-associated β-galactosidase staining solution (1 mg/mL X-gal in 40 mM citric acid/sodium phosphate, pH 6.0, 5 mM potassium ferricyanide, 5 mM potassium ferrocyanide, 150 mM sodium chloride, 2 mM magnesium chloride) at 37 °C. Post staining, the staining solution was removed and cells were stored in PBS. Senescent cells showed marked perinuclear blue staining and non-senescent cells did not exhibit this stain. A standard light microscope was used to count the number of senescence-associated β-galactosidase positive cells and the total number of cells over 5 microscope fields per sample. The percent senescence was calculated by dividing the average number of senescence-associated β-galactosidase positive cells by the average number of total cells and multiplying by 100.

### 2.10. Immunoblotting

To examine the effects of ponatinib on the levels and activity of specific marker for endothelial function such as phosphorylated eNOS, specific markers for cell survival, apoptosis and senescence such as phosphorylated AKT, cleaved caspase-3 and p16Ink, respectively, and proatherogenic molecules such as VCAM-1, ICAM-1 and HUVECs were treated with 1.7 nM of ponatinib or DMSO or 1.7 nM of ponatinib + an anti-Notch-1 antibody for 17 h and analyzed by using immunoblotting with specific antibodies. Accordingly, total proteins were isolated in an ice-cold RadioImmuno Precipitation Assay (RIPA), separated under reducing conditions and electroblotted onto polyvinylidene fluoride membrane (Immobilon-P, Millipore, Bedford, MA, USA). After blocking, the membranes were incubated overnight at 4 °C with the following primary antibodies: (1) Ser1146-phosphorylated and constitutive eNOS (Cell Signaling, Danvers, MA, USA), (2) cleaved caspase-3 (Cell Signaling), (3) phosphorylated isoform of AKT (Cell Signaling), (4) p16Ink (Cell Signaling), (5) ICAM-1 (Santa Cruz Biotechnology, Santa Cruz, CA, USA), (6) VCAM-1 (Santa Cruz). Equal loading/equal protein transfer were verified by stripping and reprobing the blots with anti-beta actin (Sigma Aldrich, St. Louis, MI, USA).

### 2.11. Statistical Analysis 

Data are expressed as mean ± standard deviation (SD). Two group comparisons were performed by using the Student *t* test for unpaired values. Multiple-group comparisons were performed by using analysis of variance and the Gabriel or Tukey Honestly Significant Difference (HSD) post-hoc test to determine statistical significance within and between groups. *p* values less than 0.05 were considered statistically significant.

## 3. Results

### 3.1. Ponatinib Reduces the Viability of Endothelial Cell and Peripheral Blood Mononuclear Cells

**Figure 1:**
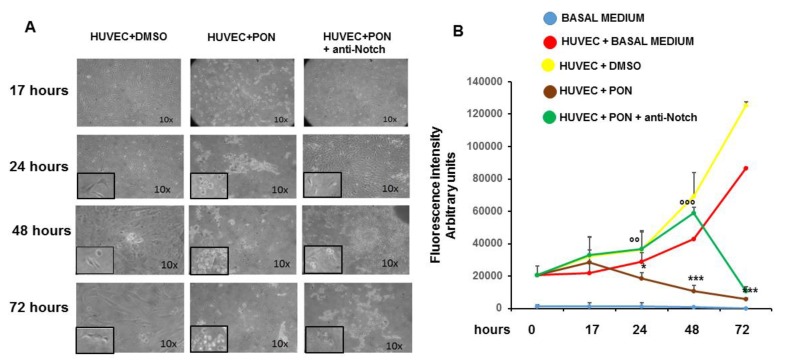
Effect of ponatinib and Notch-1 signaling inhibition on endothelial proliferation. Human umbilical endothelial cells (HUVECs) were plated at densities of 15,000 per well in 96-well plates and treated with 1.7 nM ponatinib or Dimethyl Sulfoxide (DMSO) for 17 to 72 h, in the presence or absence of 1 μg/mL of neutralizing factor anti-Notch-1 antibody. At the end of each time point, fluorescence intensities were measured with a fluorescence microplate reader using excitation at 485 nm and fluorescence detection at 530 nm. Representative microphotographs showing the effect of ponatinib and Notch-1 signaling inhibition on endothelial proliferation at 10× magnitude, are shown in Panel (**A**). Insets show the cells at 20× magnitude. Quantitative data (panel (**B**)) are presented as mean ± standard deviation of fluorescence intensity arbitrary units. *n* = 3 independent experiments, with at least 7 replicates (culture dishes) per each group. * *p* < 0.05, *** *p* < 0.001 ponatinib treated vs. control (DMSO); °° *p* < 0.01, °°° *p* < 0.001 ponatinib + anti-Notch-1 antibody treated vs. ponatinib treated. Abbreviations: Pon, ponatinib; DMSO, dimethyl sulfoxide.

Dose- and time-response proliferation curves were first performed in HUVECs and PBMNC in order to identify the highest drug concentration (1.7 nM corresponding to clinically used oral doses of 45 mg), compatible with cell maintenance in a cell cycle. Specifically, in preliminary experiments, we had verified the proliferation curve of HUVECs using various concentrations (1.7 nM, 17 nM, 170 nM). Ponatinib exerted strong cytotoxicity in HUVECs at concentrations of 17 nM and 170 nM and we achieved almost total detachment of cells from the culture monolayer to the extent that we were unable to perform all experiments with ponatinib at the highest concentrations (17 nM and 170 nM). In contrast, we could perform experiments with ponatinib at concentrations of 1.7 nM (which corresponds to clinically used oral dose of 45 mg in CML patients), including the time-dependent proliferation curve shown in Figure 1A,B.

Although HUVECs treated with 1.7 nM of ponatinib showed signs of cellular distress compared to vehicle (DMSO)-treated cells already after 17 h of incubation, the analysis of cell proliferation showed no significant differences in the incorporation rate of CyQUANTR NF fluorochrome, suggesting the maintenance of cells in the cell cycle at 1.7 nM of ponatinib (Figure 1A,B). On the contrary, the proliferation curves of PBMNCs treated with 1.7 nM of ponatinib showed an almost immediate toxicity of the drug, in terms of cell morphology, cell detachment from culture monolayer and block of the incorporation of the fluorochrome, compared to PBMNC treated with vehicle, suggesting a greater toxicity of ponatinib in this type of cells (data not shown). At 24 and 48 h, the HUVECs treated with 1.7 nM of ponatinib showed a significant reduction in the fluorochrome incorporation rate compared to DMSO, and a worsening of the morphological signs of cell suffering, suggesting the block of cell proliferation and the appearance of frank cytotoxicity of the drug. These effects were reverted by the co-incubation of the cells with 1 μg/mL neutralizing factor anti-Notch-1 antibody, suggesting that ponatinib acts on HUVECs via Notch-1 and the blocking of this signaling pathway can revert the endothelial drug toxicity (Figure 1A,B). After 72 h of treatment, HUVECs showed a complete and irreversible block of cell proliferation, which could not be reversed by the Notch-1 receptor blockage, suggesting the appearance of nonspecific cytotoxicity by ponatinib (Figure 1A,B). These results show the concentration-dependent effects of ponatinib on endothelial cell viability and greater sensitivity of PBMNC to ponatinib compared to HUVECs.

Proteomics analysis reveals the up regulation of apoptosis and the downregulation of angiogenesis and vascular development in endothelial cells exposed to ponatinib.

**Figure A1:**
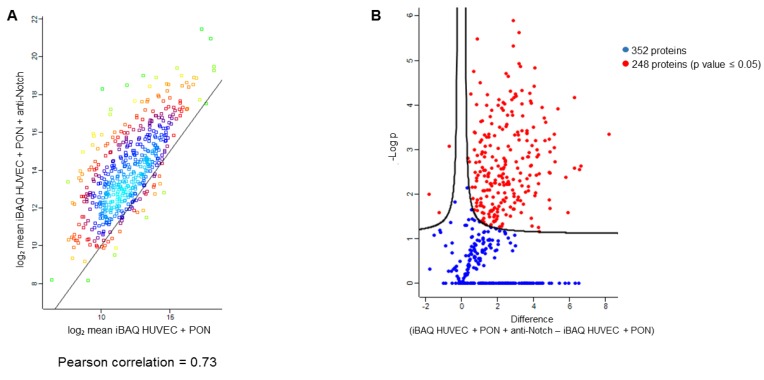
Volcano plot showing the proteins that are differentially expressed in the HUVECs treated with 1.7 nM ponatinib in the presence or absence of 1 μg/mL neutralizing factor anti-Notch-1 antibody. (**A**) Density plot showing mean Pearson correlation of average iBAQ between the two cellular treatments. (**B**) Dot plot representing the proteins differentially expressed in the two cellular treatments.

Label free proteomics analysis was carried out in HUVECs to identify the expressional signatures of endothelial cells exposed to ponatinib. In particular, quantitative proteomics data were obtained through MaxQuant software. The intensity-based absolute quantification (iBAQ) value of individual protein detected was used as a quantification parameter. The comparison of the average iBAQ of two cellular treatments revealed a mean Pearson correlation of 0.73, as shown in the density plot of Appendix A (Figure A1A).

**Figure 2:**
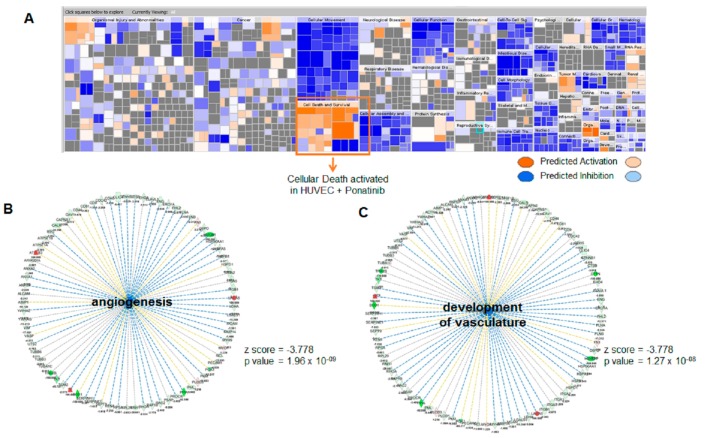
Comparative proteomic analysis of endothelial cells exposed to ponatinib and Notch-1 signaling inhibition. Panel (**A**), Functions and diseases categories activated and/or inhibited in human umbilical endothelial cells (HUVECs) treated with ponatinib compared to those co-treated with ponatinib and anti-Nothch-1 antibody are shown as a heatmap visualization, in which orange and blue shapes represent predicted activation or inhibition, respectively. IPA generates a parameter (z-score) that is able to perform a consistent prediction for activation or inhibition of such categories with statistical significance. In particular, z-scores > 2.0 indicate that a molecule and/or function is activated, whereas z-scores < −2.0 indicate the inhibition of target. Panels (**B**,**C**), downstream network analysis of the proteins from HUVECs exposed to ponatinib. Images show “angiogenesis” (**B**) and “vascular development” (**C**) categories predicted to be inhibited by the proteins from HUVECs treated with ponatinib. For both functions, the *p* value and z-score are reported.

As reported in the section of the methods, the database searching of the tandem mass spectrometry (MS/MS) data allowed us to identify and quantify in at least two analytical replicates for each condition 520 proteins for HUVECs treated with 1.7 nM ponatinib and 583 proteins for HUVECs co-treated with 1.7 nM ponatinib and 1 μg/mL anti-Notch-1 antibody. The list of quantified proteins is reported in Supplementary Materials Table S1. Moreover, the statistical analysis of all the measured proteins revealed that 248 proteins are differentially expressed in the two different experimental conditions (Appendix A Figure A1B). As reported in the volcano plot, three of these proteins were significantly up-regulated in HUVECs treated with ponatinib. In contrast, 245 proteins were significantly down-regulated in the same experimental condition (*p* value < 0.05). Quantitative raw data were compared by Ingenuity Pathway Analysis (IPA), uploading proteins fold change (HUVECs treated with ponatinib versus HUVECs co-treated with ponatinib and anti-Notch-1 antibody) for Core Analysis. Activated and inhibited downstream in HUVECs treated with ponatinib are depicted in Figure 2A as a heatmap visualization, in which the orange and blue shapes represent predicted activation or inhibition, respectively.

**Figure 3:**
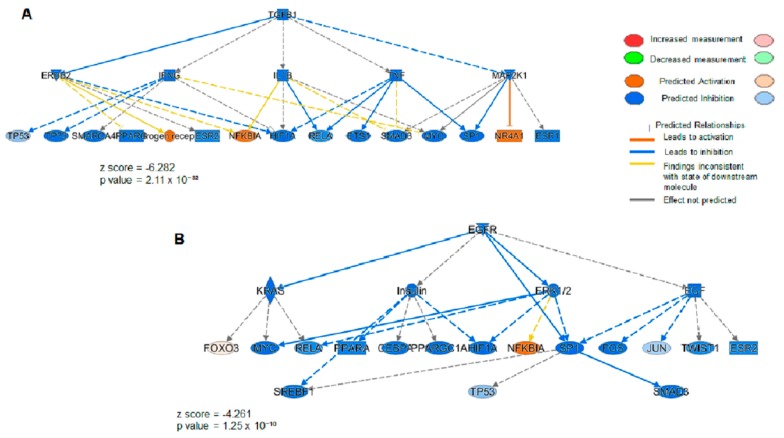
Upstream network analysis of the proteins from endothelial cells exposed to ponatinib and Notch-1 signaling inhibition. Upstream inhibited in HUVECs treated with ponatinib compared to those co-treated with ponatinib and anti-Notch-1 antibody. Panel (**A**,**B**), image shows the upstream activation of NF-kappa-B inhibitor alpha (NFKBIA) gene resulting in a z-score of 2.546, and the upstream inhibition of Transforming Growth Factor-Beta1 (TGFB1) gene (**A**) and Epidermal Growth Factor Receptor (EGFR) gene (**B**) resulting in a z-score of −6.282 and −4.261, respectively. Blue color key indicates inhibited regulators, while the orange one indicates the activated regulators. The intensity of the color is proportional to the score prediction value. As reported previously, z-scores > 2.0 indicate that a molecule and/or function is activated, whereas z-scores < −2.0 indicate the inhibition of the target.

These data show the “Functions and diseases” categories activated and/or inhibited in HUVECs treated with ponatinib in respect to those co-treated with ponatinib and anti-Notch-1 antibody. Protein cargo of HUVECs treated with ponatinib is able to trigger more certain cellular processes, particularly ones linked to “apoptosis” (*p* value = 1.24 × 10^−37^, z-score = 4.678) and “necrosis” (*p* value = 7.24 × 10^−54^, z-score = 5.932). Moreover, as shown in Figure 2B,C, the protein cargo of HUVECs treated with ponatinib is more able to inhibit “angiogenesis” (*p* value =1.96 × 10^−09^, z-score = −3.778) and “vasculature development” (*p* value =1.27 × 10^−08^, z-score = −3.778). These data show ponatinib vascular toxicity, which could be reverted by the Notch-1 receptor blockage. The differential proteins obtained from HUVECs treated with ponatinib compared to those co-treated with ponatinib and anti-Notch-1 antibody were also used for Upstream Regulator Analysis by IPA. The most significant were Transforming Growth Factor-Beta1 (TGFB1) gene (*p* value = 2.11 × 10^−32^, z-score = −6.282), Epidermal growth factor receptor (EGFR) gene (*p* value = 1.25 × 10^−10^, z-score = −4.261) and NF-kappa-B inhibitor alpha (NFKBIA) gene (*p* value = 1.02 × 10^−6^, z-score = 2.925). As reported in the mechanistic networks in Figure 3A,B, the inhibition of TGFB1 and EGFR in HUVECs treated with ponatinib compared to HUVECs co-treated with ponatinib and anti-Notch-1 antibody are directly related to each other.

These data could further confirm that ponatinib acts on HUVECs via the Notch-1 signaling pathway and that both TGFB1 and EGFR and NFKBIA are involved in the negative regulation of this pathway.

### 3.2. Ponatinib Decreases Pro-Angiogenic Endothelial Function In Vitro

**Figure 4:**
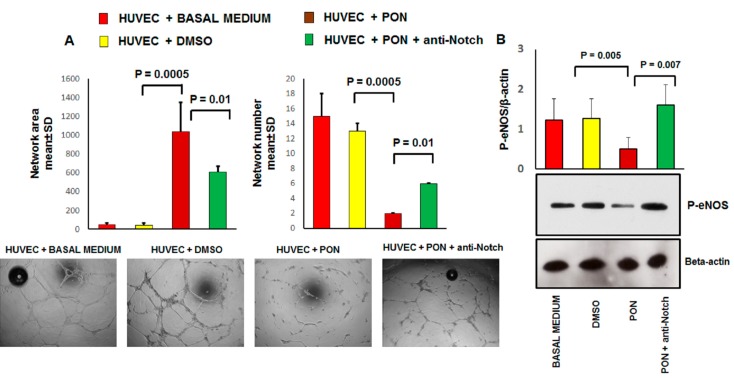
The effect of ponatinib and Notch-1 signaling inhibition on tube formation and endothelial function. Panel (**A**), Human umbilical endothelial cells (HUVECs) were plated on matrigel-coated plates and treated with 1.7 nM of ponatinib or DMSO, in the presence or absence of 1 μg/mL neutralizing factor anti-Notch-1 antibody. Tube formation was evaluated after 17 h by using light microscopy. Shown are representative fields (5× magnification) of phase-contrast. The pictures are from one representative experiment. Quantitative data (panel (**A**)) are presented as mean ± standard deviation of several parameters of tube formation. *n* = 3 independent experiments, with at least 7 replicates per each group. Panel (**B**), western analysis of phosphorylated eNOS expression in HUVECs treated with 1.7 nM ponatinib or DMSO, in the presence or absence of 1 μg/mL of neutralizing factor anti-Notch-1 antibody, with β-actin serving as a loading control. The bar graph represents for each value the mean ± S.D. from 3 separate experiments. Abbreviations: Pon, ponatinib; DMSO, dimethyl sulfoxide.

We validated the proteomics data of the effect of ponatinib on pro-angiogenic activities of endothelial cells in vitro by examining the tube formation activity using matrigel-based assay. HUVECs seeded on matrigel, formed robust tubular-like structures in the presence or absence of DMSO. 1.7 nM ponatinib dramatically inhibited HUVEC tube formation (Figure 4A).

Specifically, tubular morphogenesis was significantly abrogated 17 h after ponatinib treatment, as evidenced by a significant reduction in mean tube number, segment length, and number of junctions. These effects were partially reverted by co-incubation of the cells with 1.7 nM ponatinib and 1 μg/mL neutralizing factor anti-Notch-1 antibody (Figure 4A). Angiogenesis and vasculogenesis involve various angiogenic growth factors such as vascular endothelial growth factor (VEGF), fibroblast growth factor (FGF), angiopoietins, platelet-derived growth factors (PDGF), and signaling molecules (e.g., nitric oxide). In particular, eNOS catalyzes the conversion of l-arginine to l-citrulline generating nitric oxide (NO), which is important for the angiogenic activity of several factors including VEGF. Therefore we evaluated the impact of ponatinib on the expression of the phosphorylated isoform of eNOS (P-eNOS, the active isoform of eNOS) in HUVECs (Figure 4B). Compared to the DMSO-treated cells, HUVECs treated with 1.7 nM ponatinib showed a significant reduction in the expression of activated P-eNOS. These effects were reverted by the co-incubation of cells with 1.7 nM ponatinib and 1 μg/mL neutralizing factor anti-Notch-1 antibody, suggesting that ponatinib acts on eNOS via the Notch-1 signaling pathway (Figure 4B).

**Figure 5:**
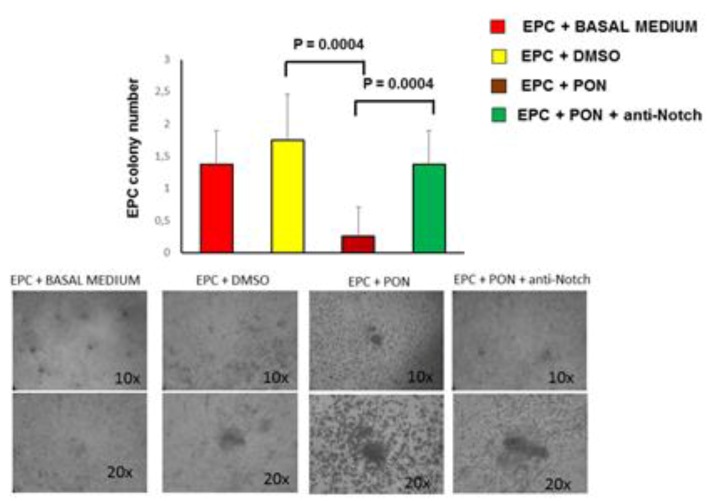
Effect of ponatinib and Notch-1 signaling inhibition on EPCs functional properties. Peripheral blood mononuclear cells (PBMCs) were plated on fibronectin-coated 24-well plates and treated, after 4 days, with 1.7 nM of ponatinib or DMSO, in the presence or absence of 1 μg/mL neutralizing factor anti-Notch-1 antibody. After 17 h of treatment, the number of colonies per field in endothelial progenitor cells (EPCs) cultured with or without ponatinib was evaluated by an inverted microscope. Shown are representative fields (10× and 20× magnification) of phase contrast. The pictures are from one representative experiment. The graph, showing number of colonies per field, represents quantification of three experiments ± standard deviation. Abbreviations: Pon, ponatinib; DMSO, dimethyl sulfoxide.

Next, we evaluated the impact of ponatinib on proliferation and functional activities of EPCs, shown to participate in the neovascularization process [21]. Compared to DMSO-treated cells, EPCs treated with 1.7 nM ponatinib for 17 h underwent a significant reduction in the capacity to form CFU-Hill early colonies (Figure 5).

These effects were reverted by co-incubation of cells with 1.7 nM ponatinib and 1 μg/mL neutralizing factor anti-Notch-1 antibody, showing that ponatinib acts on EPCs via Notch-1 signaling pathway (Figure 5). Altogether these data demonstrate that ponatinib inhibits the HUVECs and EPC pro-angiogenic activities through the Notch-1 signaling pathway.

### 3.3. Ponatinib Induces Endothelial Senescence and Apoptosis and Reduces Endothelial Survival

**Figure 6:**
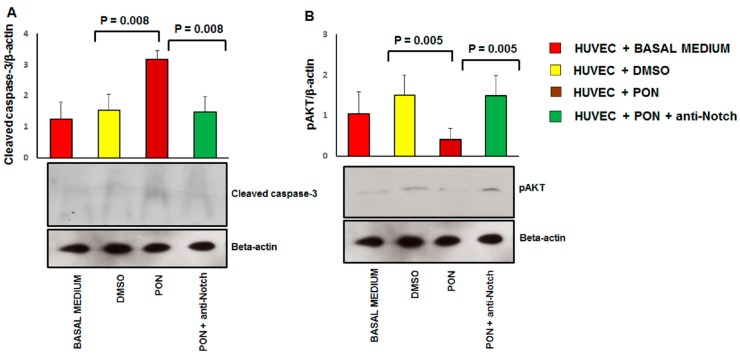
Effect of ponatinib and Notch-1 signaling inhibition on apoptosis and cell survival. Western analysis of cleaved caspase-3 (Panel (**A**)) and phosphorylated AKT (Panel (**B**)) expression in HUVECs treated with 1.7 nM of ponatinib or DMSO, in the presence or absence of 1 μg/mL neutralizing factor anti-Notch-1 antibody, with β-actin serving as a loading control. The bar graph represents for each value the mean ± S.D. from 3 separate experiments. Abbreviations: Pon, ponatinib; DMSO, dimethyl sulfoxide.

We validated the proteomics data of the effect of ponatinib on endothelial cell apoptosis and survival by examining the expression of cleaved caspase-3 and phoshorylated AKT using immunoblotting (Figure 5). Compared to the DMSO-treated cells, HUVECs treated with 1.7 nM ponatinib had a significant increase in the expression of the cleaved caspase-3 (Figure 6A) and a lower expression of the activated pAKT isoform (Figure 6B).

**Figure 7:**
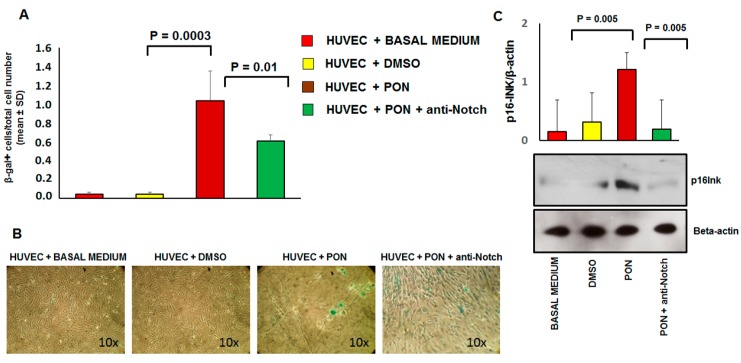
Effect of ponatinib and Notch-1 signaling inhibition on endothelial senescence. Panel (**A**), Quantitation of senescent cells in multiple microscope fields from HUVECs treated with 1.7 nM of ponatinib or DMSO, in the presence or absence of 1 μg/mL of neutralizing factor anti-Notch-1 antibody. The graph, showing the ratio between the number of senescence-associated β-galactosidase positive cells and the total number of cells over 5 microscope fields per sample, represents quantification of three experiments ± standard deviation. Panel (**B**), representative SA-β-gal staining images of HUVECs treated with 1.7 nM of ponatinib or DMSO, in the presence or absence of 1 μg/mL neutralizing factor anti-Notch-1 antibody, as indicated by blue staining. All images are shown at 10× magnification. Panel (**C**), Western analysis of senescence marker p16INK4a in HUVECs treated with 1.7 nM of ponatinib or DMSO, in the presence or absence of 1 μg/mL of neutralizing factor anti-Notch-1 antibody, with β-actin serving as a loading control. The bar graph represents for each value the mean ± S.D. from 3 separate experiments. Abbreviations: Pon, ponatinib; DMSO, dimethyl sulfoxide.

These effects were reverted by co-incubation of cells with 1.7 nM of ponatinib and 1 μg/mL of neutralizing factor anti-Notch-1 antibody, suggesting that ponatinib acts on endothelial apoptosis and cell survival via the Notch-1 signaling pathway. Senescence was identified by the combined expression of senescence-associated β-galactosidase (SA β-gal) and p16^INK4a^ (Figure 7) and reduced cell proliferation (Figure 1A,B).

**Figure 8:**
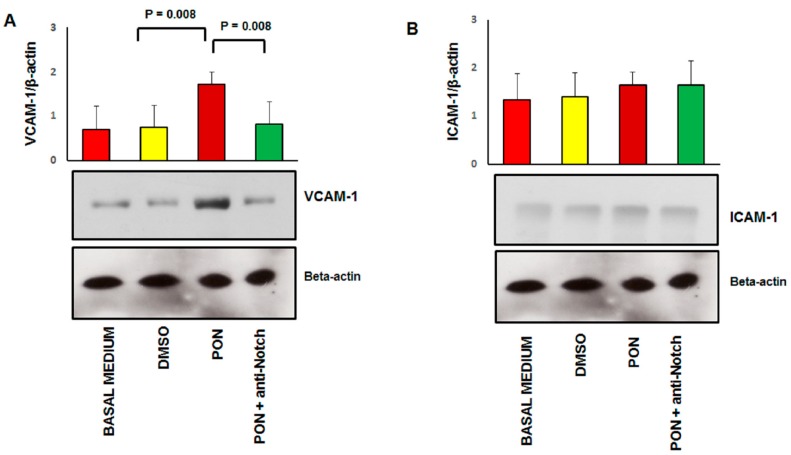
Effect of ponatinib and Notch-1 signaling inhibition on early endothalial activation markers. Western analysis of vascular cell adhesion molecule (VCAM-1, Panel (**A**)) and intercellular adhesion molecular (ICAM-1, Panel (**B**)) expression in HUVECs treated with 1.7 nM of ponatinib or DMSO, in the presence or absence of 1 μg/mL of neutralizing factor anti-Notch-1 antibody, with β-actin serving as a loading control. The bar graph represents for each value the mean ± S.D. from 3 separate experiments. Abbreviations: Pon, ponatinib; DMSO, dimethyl sulfoxide.

Specifically, SA β-gal characterizes senescence because in this condition the lysosomal content increases and has residual activity at suboptimal pH 6 [22]. As shown in Figure 7, positivity for SA β-gal (panel A) and expression of p16^INK4a^ (panel B) is significantly increased in HUVECs treated with 1.7 nM of ponatinib compared to DMSO, while proliferation was inhibited under these culture conditions (Figure 1A,B). Morphologically, HUVECs treated with ponatinib displayed rounded and flattened cytoplasm with a ‘fried egg’ appearance (Figure 1A,B). These effects were partially reverted by co-incubation of cells with 1.7 nM ponatinib and 1 μg/mL of neutralizing factor anti-Notch-1 antibody, suggesting that ponatinib acts on endothelial senescence via the Notch-1 signaling pathway, although not solely through this signaling pathway. Senescent phenotypic changes were associated with the up-regulation of VCAM-1 (Figure 8A) while the effect on ICAM-1 was not significant (Figure 8B). The effect on VCAM-1 was reverted by the co-incubation of cells with the anti-Notch-1 antibody, and in line with previous evidence of such senescence is mechanistically linked to the dysfunctional activation of endothelial cells [23].

## 4. Discussion

In this study, we demonstrate that ponatinib causes dose-dependent endothelial dysfunction, in terms of reduced angiogenic activity and increased expression of VCAM-1, one of the mediators that play a key role in the early stages of atherogenesis. Accelerated atherosclerosis may represent an additional mechanism for ponatinib-induced VAEs, as supported by clinical and in vitro studies [24]. The antiangiogenic effect of ponatinib along with the down-regulation of p-eNOS may explain several well-documented class effects of TKIs such as hypertension [25], thromboembolism [26] and left ventricular dysfunction [27]. In our study, ponatinib caused reduction of endothelial viability secondary to apoptosis and senescence of endothelial cells and down-regulation of AKT signaling pathway. Finally, ponatinib induced a dysregulation of EPCs, suggesting an imbalance between vascular damage and repair as additional mechanism explaining vascular toxicity. Here, a non-hypothesis-driven global approach was undertaken through the integration of unbiased proteomic methodologies, to profile the alterations of molecular events of HUVECs exposed to ponatinib. This approach allowed us to identify multiple signaling networks activated in ponatinib-induced vascular toxicity, and to better understand biochemical alterations in in vitro experimental conditions that mimic ponatinib-induced vascular toxicity. To the best of our knowledge, the present study is the first to use proteomics for the profiling of cellular signaling in HUVECs under ponatinib treatment. The omics analysis provided a detailed profile of changes at the molecular level and a number of altered proteins and biological functions. Specifically, the altered AKT/eNOS pathway observed in our cellular model suggests a reduction in survival and impairment of endothelial function in HUVECs treated with ponatinib, based on their inability to produce NO.

Our findings of the effects of ponatinib on endothelial cells are consistent with previous reports. Gover-Proaktor et al. reported that ponatinib reduces the viability, migration and function of human endothelial cells through inhibition of the vascular endothelial growth factor receptor [28]. In our study we also demonstrated that ponatinib exerts its endothelial effect by blunting the essential endothelial pro-survival signaling pathway AKT through the Notch-1 pathway. Notch-1 inhibition prevented endothelial apoptosis and senescence. Based on our findings, we hypothesize that pharmacological inhibition of Notch-1 signaling would protect against ponatinib-induced vascular toxicity. As proof of concept, we used the neutralizing factor anti-Notch-1 antibody to silence Notch-1. Indeed, this prevented the activation of endothelial cell death and restored phosphorylation of AKT and eNOS and endothelial viability after treatment with ponatinib.

Angiogenesis inhibitors such as tyrosine kinase inhibitors (sunitinib, ponatinib) act very effectively on the tumors and the vascular system inducing vascular dysfunction (ischemia, hypertension), so that they can present “on-target effects” on the tumor and “on-target off tumor effect” on the vessels. Notch-1 signaling plays a tumor suppressive role in chronic myeloid leukemia (CML) [29,30]. Overexpression of the active form of Notch1 or Notch2 in K562 cells has been reported to inhibit proliferation, accompanied by an increase in Hes1 mRNA levels [29]. Therefore, specific activation of Notch signaling in CML cells by ponatinib can be considered as the “on-target effect” on the tumor and represents a therapeutic approach for CML. Notch also plays a central role in cell fate decisions, including vascular development [17]. However, hyperactivation of Notch-1 in the vessels can lead to abnormal vascular development and vascular dysfunction [30]. Therefore, by hyperactivating Notch-1 in the vessels, ponatinib exerts an “on-target off tumor effect”, which leads to deleterious effects and may explain the drug’s vasculotoxicity. Our data have shown that inhibition of the ponatinib “on target off tumor” effect through blockade of the Notch receptor leads to reversal of the vasculotoxicity of ponatinib. Therefore, strategies for endothelium-specific inhibition of Notch-1 such as anti-Notch antibodies [31], are warranted in order to protect the endothelium from ponatinib-induced vascular toxicity, without interfering with the anticancer effect of the drug.

## 5. Conclusions

We have here demonstrated that ponatinib significantly increased endothelial toxicity in vitro. Importantly, we have identified the AKT/eNOS and Notch-1 pathways as key targets of ponatinib. We have shown that the Notch-1 pathway likely mediates, at least in part, the vascular toxicity associated with this agent.

## Supplementary Materials

The following are available online at https://www.mdpi.com/2077-0383/9/3/820/s1, Table S1: list of quantified proteins.

## Abbreviations

Bovine Serum Albumin	BSA
Chronic myeloid leukemia	CML
Colony forming units-endothelial cells	CFU-ECs
Coronary artery disease	CAD
Dimethyl sulfoxide	DMSO
Endothelial progenitor cells	EPCs
Endothelial nitric oxide kinase	eNOS
Epidermal growth factor receptor	EGFR
Fibroblast growth factor	FGF
Filter Aided Sample Preparation	FASP
Human umbilical endothelial cells	HUVECs
Ice-cold RadioImmuno Precipitation Assay	RIPA
Ingenuity Pathway Analysis	IPA
Intensity-based absolute quantification	iBAQ
Intercellular adhesion molecular	ICAM-1
Liquid chromatography tandem mass spectrometry	LC-MS/MS
NF-kappa-B inhibitor alpha	NFKBIA
Nitric oxide	NO
Peripheral arterial obstructive disease	PAOD
Peripheral blood	PB
Peripheral blood mononuclear cells	PBMNCs
Phosphate Buffered Saline	PBS
Platelet-derived growth factors	PDGF
Senescence-associated β-galactosidase	SA β-gal
Transforming Growth Factor-Beta1	TGFB1
Tyrosine kinase inhibitor	TKI
Vascular endothelial growth factor	VEGF
Vascular adverse events	VAEs
Vascular cell adhesion molecule	VCAM-1
